# Protein interaction networks provide insight into fetal origins of chronic obstructive pulmonary disease

**DOI:** 10.1186/s12931-022-01963-5

**Published:** 2022-03-24

**Authors:** Annika Röhl, Seung Han Baek, Priyadarshini Kachroo, Jarrett D. Morrow, Kelan Tantisira, Edwin K. Silverman, Scott T. Weiss, Amitabh Sharma, Kimberly Glass, Dawn L. DeMeo

**Affiliations:** 1 Channing Division of Network Medicine, Department of Medicine, Brigham and Women’s Hospital, Harvard Medical School, Boston, MA 02115 USA; 2 Division of Pediatric Respiratory Medicine, University of California San Diego, San Diego, USA; 3 Division of Pulmonary and Critical Care Medicine, Brigham and Women’s Hospital, Boston, MA USA; 4 Center for Complex Network Research, Northeastern University, Boston, MA USA

**Keywords:** COPD, AGE-RAGE pathway, Protein–protein interaction networks, Methylation

## Abstract

**Background:**

Chronic obstructive pulmonary disease (COPD) is a leading cause of death in adults that may have origins in early lung development. It is a complex disease, influenced by multiple factors including genetic variants and environmental factors. Maternal smoking during pregnancy may influence the risk for diseases during adulthood, potentially through epigenetic modifications including methylation.

**Methods:**

In this work, we explore the fetal origins of COPD by utilizing lung DNA methylation marks associated with in utero smoke (IUS) exposure, and evaluate the network relationships between methylomic and transcriptomic signatures associated with adult lung tissue from former smokers with and without COPD. To identify potential pathobiological mechanisms that may link fetal lung, smoke exposure and adult lung disease, we study the interactions (physical and functional) of identified genes using protein–protein interaction networks.

**Results:**

We build IUS-exposure and COPD modules, which identify connected subnetworks linking fetal lung smoke exposure to adult COPD. Studying the relationships and connectivity among the different modules for fetal smoke exposure and adult COPD, we identify enriched pathways, including the AGE-RAGE and focal adhesion pathways.

**Conclusions:**

The modules identified in our analysis add new and potentially important insights to understanding the early life molecular perturbations related to the pathogenesis of COPD. We identify AGE-RAGE and focal adhesion as two biologically plausible pathways that may reveal lung developmental contributions to COPD. We were not only able to identify meaningful modules but were also able to study interconnections between smoke exposure and lung disease, augmenting our knowledge about the fetal origins of COPD.

**Supplementary Information:**

The online version contains supplementary material available at 10.1186/s12931-022-01963-5.

## Background

Chronic obstructive pulmonary disease (COPD) is a leading cause of death worldwide [1–3] and may be diagnosed in adults reporting a history of childhood asthma and maternal smoke exposure [4–8]. It is a complex disease, influenced by multiple factors including genetic variants, and environmental factors, including exposure to maternal smoking in early fetal life and personal smoking in later life. Maternal smoking during pregnancy may influence the risk for diseases during adulthood, potentially through epigenetic modifications including methylation [9–13]. Primary prevention of adult lung diseases includes identifying predisposing molecular factors [14, 15].

Recent observations support that genes associated with complex traits have protein products that tend to interact with each other more frequently than expected by chance [16–22]. Therefore, a single gene does not function as a single activator for a disease, but the interplay of multiple genes will eventually lead to a pathogenesis [22–24, 40]. Network-based approaches can be used to identify these groups of genes. Genes associated with an exposure or disease may form connected subnetworks (exposure or disease modules containing usually 10 to 100 genes) within the larger protein–protein interaction network (PPI). Furthermore, genes in close proximity in the PPI annotate to similar functional pathways. Network-based approaches for studying complex diseases have identified COPD disease modules [25–33]. Most approaches use methods which are based on seed genes, sets of 5–30 genes associated with a disease such as COPD that are used as a starting set, with additional genes added to the module iteratively based on the topology of the network [25, 27, 30, 34]. Other methods use similarity measures between transcriptomic data [26, 28, 29, 33] and most studies highlight a single module only. However, some identify additional modules associated with respiratory diseases [25, 27, 29] and analyze the interactions and linking molecular mechanisms between the different modules. Typically, only one omic data type has been used, usually transcriptomic data.

In this current work, to identify network modules related to IUS-exposure and adult lung disease, we compute significantly connected components using DNA methylation and gene expression association information from lung tissue and a functional PPI [35]. For fetal and adult lung methylation and adult lung expression data, genes were selected based on at least nominal statistical thresholds for association with IUS-exposure and COPD, respectively.

We identified network modules and studied the connectivity between the fetal lung DNA methylation and COPD DNA methylation and expression modules. Leveraging these modules, we highlight biological mechanisms and common pathways, including the AGE-RAGE pathway, which may provide molecular links between lung development and COPD.

## Materials and methods

### Data

We used published results from a fetal lung DNA methylation data set and COPD DNA methylation and expression data sets [36–38].

### Fetal lung

The fetal lung DNA samples included 78 samples that passed the quality control measures [36]. Methylation in smoke-exposed was compared to unexposed fetal lung samples and were considered nominally significant at a p-value cut off of 0.05. The fetal lung DNA samples were isolated from discarded tissue from 8–18 weeks of gestation. The samples were anonymized at study entry at the Laboratory of Developmental Biology, University of Washington, Seattle, WA, USA.

Genome-wide methylation assay was performed using 750 ng of bisulfite-treated DNA per sample using the Infinium HumanMethylation450 BeadChip array (Illumina, San Diego, CA, USA), according to manufacturer’s recommended protocol. Data were available for gestational age, fetal sex, and cotinine levels. Sex was verified using X chromosome methylation. IUS exposure was inferred by measuring placental cotinine concentrations. Exposure was treated as a continuous and dichotomous variable, with levels of cotinine ≤ 7.5 ng/g considered as unexposed (control group) and levels of cotinine > 7.5 ng/g as exposed. Published results were used from site based differential methylation analysis from limma (version 3.37.7) [39] adjusting for age, sex, sample plate, and sentrix position. DM CpG sites were nominally significant at a p-value cut off of 0.05 and mapped to genes using Human Genome build: GRCh37/hg19 annotation.

### COPD

Genome-wide methylation assay was performed using 750 ng of bisulfite-treated DNA per sample using the Infinium HumanMethylation450 BeadChip array (Illumina, San Diego, CA, USA) and gene expression was assayed using the Illumina HumanHT-12 Bead Chips [37, 38]. CpG sites were mapped to genes using Human Genome build: GRCh37/hg19 annotation.

The study included lung tissue samples from 114 COPD cases (avg. age 63.4, 60% males, all former smokers, quit smoking 84.7 months before on avg., FEV 1% predicted 26.3 avg.) and 46 control smokers with normal lung function (avg. age 65.3, 29% males, all former smokers, quit smoking 181 months before on avg., FEV 1% predicted 98.1 avg.).

Published results were used fromsite based differential methylation and gene-based expression analyses performed using limma (version 3.37.7) [39]. Previously published results [37, 38] were included at a p-value cut off of 0.05. CpG sites were mapped to genes using Human Genome build: GRCh37/hg19 annotation.

### Protein–protein interaction network

In order to find meaningful connected components, a PPI of decent size and non-sparsity is required. The predictive power of the connectivity significance increases as the PPI becomes more complete [41]. We used the HumanNet-FN [35] PPI (downloaded April 2019 https://www.inetbio.org/humannet) which includes co-functional links (given by co-essentiality, co-expression, pathway database, protein domain profile associations, gene neighborhood, and phylogenetic profile association) and protein–protein interactions (given by high-throughput assays and literature curated interactions). The network consists of 17,247 genes, which are connected by 371,502 undirected edges (where 118,012 are physical, 213,003 functional, and 39,587 are physical and functional interactions). The largest connected component (LCC) of the PPI consists of 17,191 genes which are linked to each other by 371,464 edges.

An overview of the data sets and their LCCs in the PPI can be found in Table 1.

### Computation of the modules

The method used here is an extension of the work of Wang et al [42] which selects all nominally-significant genes (p-value < 0.05) and then uses fold change values for ranking genes. The framework identifies exposure or disease modules by agglomerating genes based on their statistical significance within their respective study.

Our approach here is similar, except that it considers all genes of the data set (not only nominally-significant genes), ranking them according to their p-value (rather than fold change), from the most significant to the least significant. The remaining steps are the same as in [42]. First, different thresholds for the p-values are given. Next, for each threshold the LCC is identified which is given by all genes which have a p-value lower than the threshold. With increasing p-value thresholds the sizes of the LCCs increase. The sizes of the LCCs are then compared against random expectation and a z-score is computed to indicate their significance. Thus, we obtain a p-value threshold vs. z-score plot which is used to determine the module. The module is the LCC with a z-score above 1.6 and of a size which is in general considered to be a reasonable size for a module (30–100) containing genes which have relatively small p-values. If several LCCs match these criteria we choose the one with the highest z-score. Thus, the method ensures that the genes which can be most strongly associated with a phenotype of interest are preferentially added to the module while maintaining significant module connectivity. We provide a detailed method description in Additional file 1 (section “Computation of the modules”).

We identified one module for each methylation set (fetal lung and adult COPD) and one for the COPD gene expression set. Additionally, we computed two modules for the 502 genes found in the fetal lung and COPD sets. Here, a module was computed using the p-values given by the fetal lung methylation data set and another one was computed using the p-values given by the COPD methylation data set (Additional file 1 section “Computation of the modules using genes which are significantly enriched in both methylation data sets” and “Modules computed using genes which are significantly enriched in both methylation data sets”).

### Robustness

To study the topological robustness of the modules, we evaluated whether highlighted module genes form significantly connected components in five different PPIs (BioGRID [43], STRING [44], Hint [45], PPI2016 [46], and BioPlex [47]). To do so, we first identify the LCC given by the modules’ genes in the other PPIs and next compared this size against random expectation. All modules form significantly connected component in all five PPIs except for the COPD methylation module in the STRING PPI. These results show that the modules (and the method) are robust irrespective of the choice of PPI (Additional file 1 section “Robustness” and Additional file 2: Table S1).

### Genes associated to COPD

In order to identify genes previously associated with COPD we used the database DisGeNet [48]. We entered each gene individually and filtered the “Summary of Gene Disease Association’s” results for “Disease Classes” containing “Respiratory Tract Diseases”.

### Enrichment analyses

We performed enrichment analyses on different sets of genes given by the computed modules and their connections to the other modules. For all analyses we used g:Profiler [49] (accessed May 2020) using the 17,190 genes in the LCC in the HumanNet-FN (Additional file 3: Table S2) as background and the default parameters otherwise. We considered a pathway as significantly enriched with a p-value < 0.05. We performed an enrichment analysis for each set of genes in each module and for each set of interactors.

## Results

**Fig. 1:**
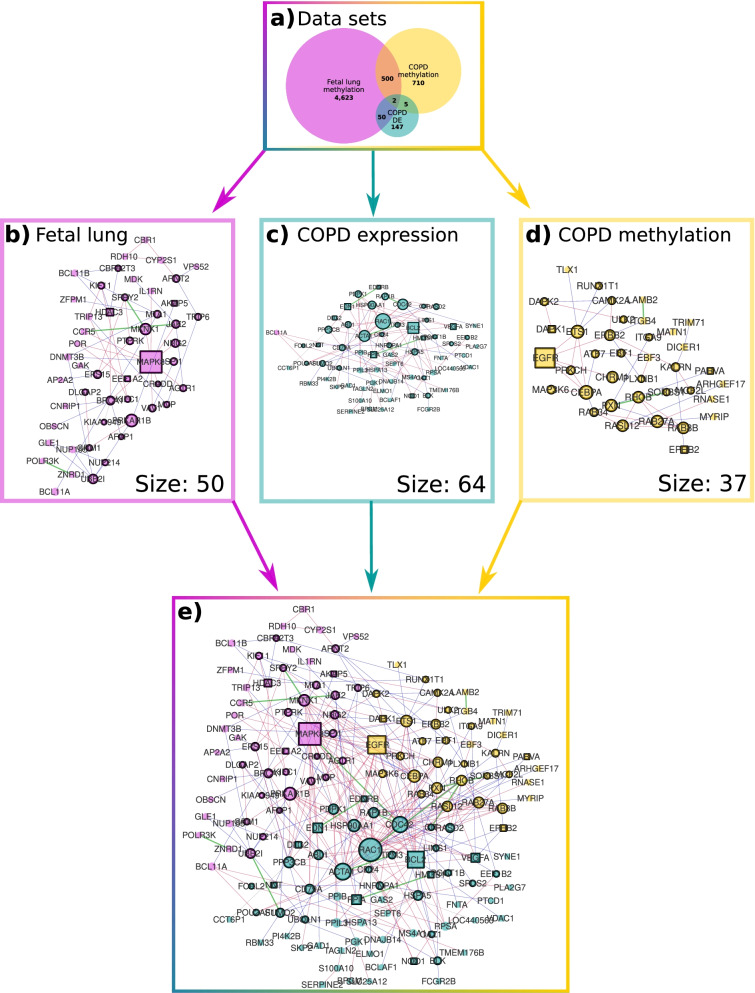
**a** Venn diagram of the three data sets: The Venn diagram shows the numbers of the genes which are mapped to nominally differentially methylated CpG sites or significantly differentially expressed in the data set and their overlap. For **b**–**e** The sizes of the genes relate to their degree within this module. Genes that are associated with COPD are represented as a square. Genes that have a heavier border connect to other modules. The blue edges represent physical interactions, the red edges functional interactions, and green edges represent both. **b** Fetal lung methylation module: The module consists of 50 significant genes from the fetal lung methylation data set. **c** COPD expression disease module: The module consists of 64 significant genes from the COPD gene expression data set. **d** COPD methylation disease module: The module consists of 37 significant genes from the COPD methylation data set. **e** The three modules form a connected component in the HumanNet-FN: The fetal lung methylation module (pink), the COPD expression disease module (turquoise), and the COPD methylation disease module (yellow) form one large connected component. The sizes of the nodes correspond to their degree within the shown component

We used published results and compared 5175 genes which were annotated to nominally differentially methylated CpG sites in the fetal lung data set [36] to the 1217 genes that were differentially methylated CpG sites and 204 genes differentially expressed in the adult COPD data set [37, 38] (Table 1 and Fig. 1a). Two genes are differentially expressed and differentially methylated in all three data sets: *ODF3L1* and *DTX1*.

We used the HumanNet-FN PPI [35] (downloaded April 2019) which includes co-functional links and protein–protein interactions. The LCC given by the genes in the fetal lung data set consists of more than 4,000 genes and the LCC given by the genes in the COPD methylation data set consists of more than 700 genes (Table 1). Most published disease modules consist of 10 to 100 genes [34, 41, 50, 51] and we therefore computed connected components of smaller size for further analyses.

We will first introduce results from the fetal and adult lung methylation and expression modules (see section “Modules”), and the interactors between these modules (see section “Interactors linking IUS-exposure and disease modules”).

### Modules

The set of 5175 genes in the fetal lung methylation data set produced an IUS-exposure module of 50 genes (Table 2). We found that 7 of the 50 genes (14%) (hypergeometric p-value = 0.04) have been related to COPD (Fig. 1b, Additional file 1 section “Fetal lung methylation module” and Table 3).

**Table 1:** For each data set we show the number of genes which could be mapped to nominally differentially methylated CpG sites, or which are differentially expressed

Data set	# Genes, which could be mapped to nominally differentially methylated CpG sites	# Genes, which could be mapped to nominally differentially methylated CpG sites, found in the PPI	Size of the LCC given by genes, which could be mapped to nominally differentially methylated CpG sites
Fetal lung methylation	5175	4599	4297
COPD methylation	1217	1037	721
COPD expression	204	181	64

“Data set”: Which data set is considered.”# Genes, which could be mapped to nominally differentially methylated CpG sites”: How many genes could be mapped to nominally differentially methylated CpG sites or are significantly differentially expressed.”# Genes, which could be mapped to nominally differentially methylated CpG sites, found in the PPI”: How many of the significant genes of the set can be found in the protein protein interaction network HumanNet-FN.”Size of the LCC given by genes, which could be mapped to nominally differentially methylated CpG sites”: Size of the largest connected component in the HumanNet-FN given by the significant genes

**Table 2:** List of genes for each module

Genes	Name of module the interactors are member of	Name of module the interactors connect to
*AGTR1, AKAP5, ARNT2, JAK2, KIF11, MAPK8, MKNK1, MTA1, NRG2, PRKAR1B, PTPRK, SPI1, SPRY2, TRIP6*	Fetal lung methylation module	COPD methylation module
*MAPK8, MKNK1, NRG2, AGTR1, CRADD, BRCA1, VAV1, JAK2, MTA1, PRKAR1B, UBE2I, NUP214, GRM1, DLGAP2, EEF1A2, AFAP1, MVP, TRIP6, PTPRK, EPS15, KIAA1949, BCL11A, KIFC1*	Fetal lung methylation module	COPD expression module
*POLR3K, VPS52, CBR1, BCL11B, OBSCN, RDH10, AP2A2, GAK, ZFPM1, ZNRD1, NUP155, IL1RN, GLE1, DNMT3B, POR, CYP2S1, TRIP13, CNRIP1, CCR5, MDK*	Fetal lung methylation module	No module
*ATF7, CAMK2A, CEBPA, CHRM1, DAPK1, DAPK2, EBF1, EGFR, ERBB2, ETS1, MAP3K6, PRKCH, PXN, RUNX1T1, ULK2*	COPD methylation module	Fetal lung methylation module
*CEBPA, CHRM1, DAPK1, EBF1, EGFR, ERBB2, ETS1, MAP3K6, PRKCH, PXN, ULK2, EPHB2, ITGA9, KALRN, MCF2L, PARVA, PLXNB1, RAB27A, RAB34, RAB8B, RASL12, RHOB, SORBS1*	COPD methylation module	COPD expression module
*TRIM71, EBF3, DICER1, MYRIP, TLX1, ARHGEF17, MATN1, LAMB2, ITGB4, RNASE1*	COPD methylation module	No module
*BLK, RASD2, CDC42, RAP1B, EDNRB, HNRNPA1, TPM3, EEF1B2, EDN1, VEGFA, BCL2, CIT, HMGB1, SPCS2, RAC1, ABL1, HSP90AA1, OAZ1, PGGT1B, CD24, ACTA1, LIMS1, PDPK1*	COPD expression module	COPD methylation module
*CDC42, NQO1, RAP1B, CD79A, ABL1, EDN1, HSP90AA1, PPP3CB, BCL2, VEGFA, ACTA1, SUMO2, PPIA, PDPK1, RAC1, HSPA5, DLG2, EEF1B2, NNT, FCRL2, UBQLN1, BLK, POU2AF1, BCL11A*	COPD expression module	Fetal lung methylation module
*TMEM176B, S100A10, BCLAF1, SEPT6, SYNE1, PGK1, HSPA13, CCT6P1, RBM33, MS4A1, PPIB, SKP2, ELMO1, SLC25A12, GAS2, PPIL3, LOC440563, FCGR2B, FNTA, TAGLN2, PTCD1, PLA2G7, VDAC1, BPGM, GAD1, DNAJB14, SERPINE2, PI4K2B, RPSA*	COPD expression module	No module

The second column gives the name of the module the genes are member of, whereas the third column gives the name of the module the genes connect to. If the third column is “No module” then those genes are not interactors

All results, including the Gene Disease Association score can be found in the Table 3 and Additional file 4: Table S3. Additionally, we looked for associations of genes to COPD according to GWAS study using the study of Sakornsakolpat et al. [52].

The COPD disease module given by the 1217 genes in the COPD methylation data set (adj. P-value < 0.05) [37] consists of 37 genes (Table 2), and 4 (11%) have prior associations to COPD (hypergeometric p-value = 0.15) (Fig. 1c, Additional file 1 section “COPD methylation disease module” and Table 3).

There are 204 genes significantly differentially expressed in the adult COPD gene expression data set (adj. p-value < 0.05) [37] and the resulting disease module consists of 64 genes (Fig. 1d, Table 2). Twelve genes of the module (19%) have prior associations with COPD (hypergeometric p-value = 0.001) (Additional file 1 section “COPD expression module” and Table 3).

### Interactors linking exposure and disease modules

**Fig. 2:**
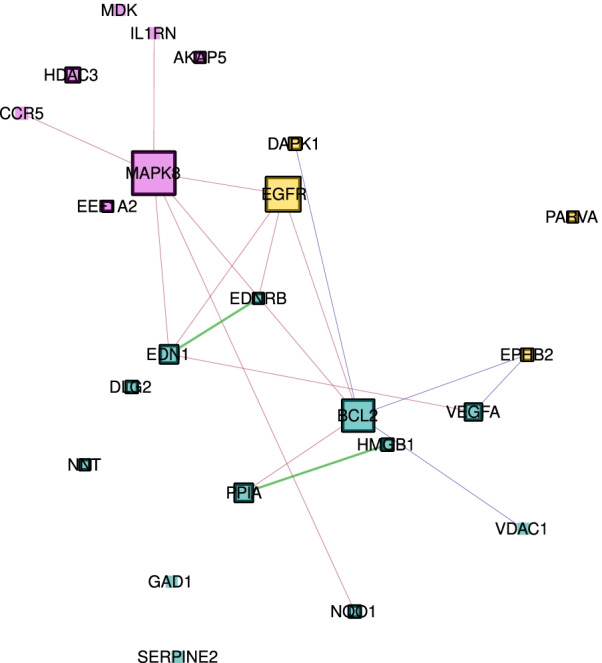
The subnetwork of the connected component in Fig. 1e given by all genes which can be associated with COPD. Using the subnetwork given by the three modules (Fig. 1e) we only kept the genes, and their interactions, which can be associated to COPD by DisGeNet or GWAS studies. Pink genes are members of the fetal lung methylation module, yellow genes are members of the COPD methylation disease module and turquoise genes are members of the COPD expression disease module. The blue vs. red, edges represent physical vs. functional, interactions, whereas green edges represent both

**Fig. 3:**
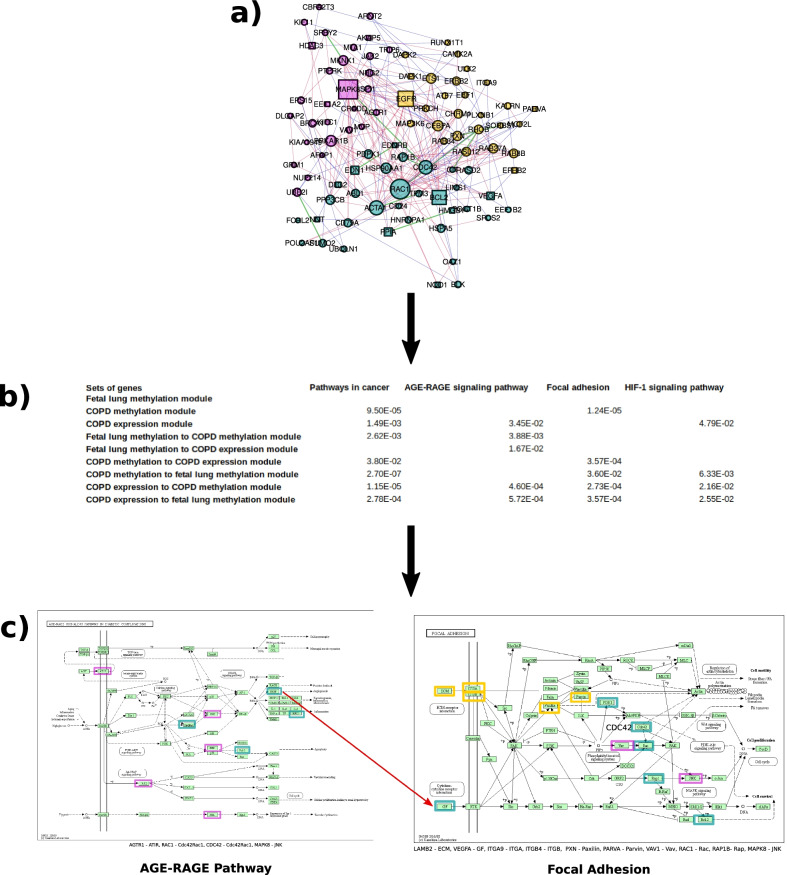
**a** The subnetwork given by all interactors. Using the subnetwork given by the three modules (Fig. 1e) we only kept the genes of the modules which are connected to another module. Pink genes are members of the fetal lung methylation module, yellow genes are members of the COPD methylation disease module and turquoise genes are members of the COPD expression disease module. The blue vs. red edges represent physical vs. functional interactions, whereas green edges represent both. **b** We show here all pathways which were significantly enriched for at least four of the sets of genes listed in the first column. The pathways are in the first row. The adj. p-value is given if the pathways were significantly enriched (adj. p-value < 0.05) using the set of genes and the KEGG data base. Module *A* to Module *B*: The set of intermediate genes from module *A* connecting to module *B* were used for the enrichment. **c** AGE-RAGE Pathway: The AGE-RAGE pathway was enriched for interacting genes between the COPD expression disease module and the fetal lung methylation module, as well as for both sets of interactors within the fetal lung methylation module and the interactors between the COPD expression and COPD methylation module. The pink squared genes are the interactors which locate in the fetal lung methylation module and the turquoise genes locate in the COPD expression disease module. Note that VEGFA is downstream in the AGE-RAGE pathway and upstream for the Focal Adhesion (red arrow) and is identified in the COPD expression disease module. Focal Adhesion Pathway: The Focal Adhesion Pathway is enriched for interacting genes between both of the COPD disease modules and the fetal lung methylation module as well as for the interactors between the COPD expression and COPD methylation module. The yellow squared genes are from the COPD methylation disease module, the turquoise genes are in the COPD expression disease module, and the pink squared genes are the interactors which locate in the fetal lung methylation module. Note that VEGFA links the AGE-RAGE pathway and the Focal Adhesion and is located in the COPD expression disease module

The three modules support genomic links between IUS-exposure and COPD in adults. The methylation modules for fetal and adult lung do not overlap and the fetal lung methylation module and the COPD expression module have only one gene in common (*BCL11A*). Therefore, we focused using our method to explore genes connecting the fetal lung IUS exposure and adult COPD PPI modules. Both COPD disease modules contain genes which are directly connected to genes of the fetal lung methylation module in the HumanNet-FN (Fig. 1e). The number of edges connecting these modules is higher than expected by chance (p-value < 1e−05) (Additional file 1 section “Connectivity between the modules”); most edges (196 out of 286, 69%) connecting the modules with each other are functional. In total there are 66 genes which connect one module with another and we call these genes interactors. Twenty-seven interactors are members of the fetal lung methylation module, of which 13 connect to the COPD methylation disease module and 23 to the COPD expression disease module (9 genes are connected to both modules) (Table 2). Fifteen genes of the COPD methylation disease module and 24 genes of the COPD expression disease module connect to the fetal lung methylation module (Figs. 1e and 3a, Tables 3 and Additional file 3: S2). Genes with prior known associations to COPD in the literature are well connected (z-score = 8.1, p-value = 1.4e−5) (Additional file 1 section “Connectivity of the genes which can be associated to asthma and/or COPD”), especially between the three modules, with predominant functional edges (hypergeometric p-value = 1.4e−05) (Fig. 2). There are in total 21 genes in the modules which can be associated with COPD. Not all of them are connected to each other, but the largest connected component contains 13 genes (Table 3). Half of the 24 interactors of the COPD expression module which are connected to the fetal lung methylation module are up-regulated while the other half is down-regulated. Sixteen out of the 23 interactors in the COPD expression module connected to the COPD methylation module are down-regulated (Additional file 5: Table S4).

**Table 3:** Each list contains the genes within the corresponding module if they can be associated to COPD according to the database DisGeNet or GWAS study

Module	List of genes which can be associated to COPD
Fetal lung methylation module	AKAP5, CCR5, EEF1A2, HDAC3, IL1RN, MAPK8, MDK
COPD methylation module	EGFR, SORBS1, DAPK1, EPHB2, PARVA
COPD expression module	BCL2, DLG2, EDN1, EDNRB, GAD1, HMGB1, NQO1, PPIA, SERPINE2, VDAC1, VEGFA

The interactors, as linking genes, are of potential interest since we hypothesize that these may capture genomic trajectories between perturbations in lung tissue during fetal development and COPD in adulthood. Therefore, the 66 interactor genes were subjected to pathway enrichment analysis to identify perturbed pathways that may mark susceptibility to COPD.

### Enrichment analysis of the interactors

**Fig. 4:**
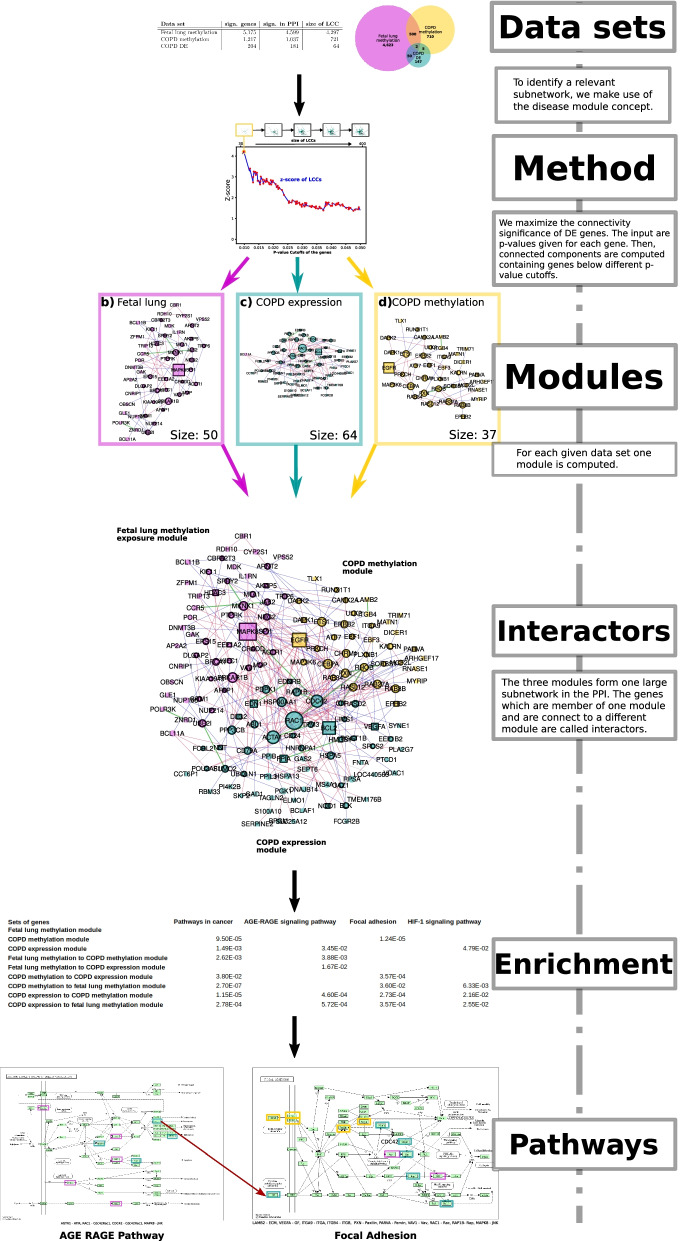
The figure is an overview of the methods and analyses performed in this study. We start with three different Data sets each providing a set of genes. Using these genes and the ENCORE Method we compute for each data set a connected component in the HumanNet PPI, a Module. Next, we analyze the connectivity of these three modules in the PPI. Doing so, we can identify Interactors, genes which link different modules with each other. Performing an Enrichment analysis on these interactors reveals different Pathways, including the AGE-RAGE pathway and Focal Adhesion. This methodological workflow can be applied to any lung disease with published results

We performed enrichment analyses on seven gene sets given by the modules and their connections (Figs. 1e, 3a), using KEGG [53], and the LCC of the HumanNet-FN as background.The results of the enrichment analyses can be found in Fig. 3 and 4, as well as in Additional file 6: Table S5. First, we performed three enrichment analyses using the whole set of genes of each module, including the fetal lung methylation module (50 genes), the COPD methylation module (37 genes), and the COPD expression module (64 genes) (Table 2).

Next, we performed an enrichment analysis for each set of interactors: the set of genes from the fetal lung methylation module which are connected to the COPD methylation module (14 genes) and the set of genes from the fetal lung methylation module which are connected to the COPD expression module (23 genes), the set of genes from the COPD methylation module which are connected to the fetal lung methylation module (15 genes), and the genes from the COPD expression module which are connected to the fetal lung methylation module (24 genes).

All significantly enriched pathways (adj. p-value < 0.05) for at least three sets of the genes defined above are listed in the table in Fig. 3b (see Additional file 6: Table S5 for more details). The pathway which was significantly enriched for most gene sets (four out of seven gene sets) was the AGE-RAGE pathway, followed by the Focal-Adhesion pathway.

## Discussion

COPD is a complex multi-factorial disease with no known cure. Understanding early life susceptibility factors, including epigenetic factors, may lead to preventative interventions [54–56]. Many studies of COPD susceptibility have focused on genetic factors, but environmental perturbations starting in utero may contribute to fetal programming and set epigenetic trajectories of lung disease [57]. In utero exposures such as cigarette smoking and perturbed lung growth and development are associated with COPD, but there are limited insights into the molecular links between early exposures, lung growth and adult disease. It is likely that in utero exposures do not impact single genes but networks of genes. Using protein–protein interaction networks to study links between smoking-related perturbations during lung development and COPD is of clinical significance as identified genes and networks may provide insights into biomarkers and targets for primary prevention of adult lung disease [58]. Prior observations linking in utero tobacco smoke with COPD support fetal programming, but mechanisms are not fully understood [59]. Here, we focus on fetal lung methylation marks associated with IUS exposure which may link to molecular signatures to adult COPD.

Simple intersections of DNA methylation associations may not reveal links between early life exposures and lung disease [36]. Here, we applied a protein–protein interaction network-based approach using published results to generate modules for fetal and adult lung tissue to link IUS-exposure and COPD susceptibility. However, the module characteristics are highly dependent on the completeness of the PPI and the data sets used. We used available PPIs to verify our results, but future work must include functional validation of network findings.

COPD heterogeneity and cellular heterogeneity in lung tissues may impact the modules characterized using bulk genomic results. The COPD lung tissue cohort has limited information regarding COPD subtypes (emphysema vs chronic bronchitis) [38]. For this manuscript, we leverage published results for COPD based on a spirometric diagnosis. Future work needs to consider subtype specific molecular associations and network models. Longitudinal birth cohorts are limited for addressing links between fetal exposures impacting lung tissue and adult lung disease, as molecular markers are generally studied using cord blood not fetal lung tissue. Leveraging life-course genomic data is also an important direction for future investigation.

There are only two genes which are significantly differentially expressed or methylated in all three data sets: *ODF3L1* (Outer Dense Fiber Of Sperm Tails 3 Like 1) and *DTX1* (Deltex E3 Ubiquitin Ligase 1). *ODF3L1* has not been studied extensively beyond associations with testis but as a class ODF proteins have been implicated in cytoskeleton pathways and cilia. *DTX1* has been implicated in Notch signaling [60] and is key ubiquitin E3 ligase implicated in multiple pathways including development [61].

The omnigenic model distinguishes between core and peripheral genes, where core genes can be strongly associated with the studied phenotypes and the peripheral genes have a small effect on disease risk. Therefore, to understand complex diseases, additional information beyond genetic variation needs to be integrated into the model. To account for this, we computed COPD modules using transcriptomics and epigenetic information. Additionally, we identified a module associated to leveraged data from IUS exposure of fetal lung. Using these three modules and their adjacency within the PPI we were able to study more than just the most significant genetic associations to COPD.

In order to identify “*core*” genes [23] we first identified a module [42] for each data set. Interestingly, the three modules do not have any genes in common, except for *BCL11A*. Thus, each module captures the associated phenotype individually [23]. To evaluate a potential link between IUS perturbed lung development and COPD we analyzed the connection of the fetal lung methylation module to the two COPD disease modules. COPD related genes connecting the modules are potentially functionally related through diverse aspects such as airway remodeling, immune response, and inflammation. The number of interactions between the three modules is higher than expected by chance suggesting that the perturbation of the genes in one module potentially impacts the functionality of the genes within the other modules. Most edges connecting the modules with each other are functional not physical interactions between proteins. Interestingly, 16 of the 23 interactors in the COPD expression module which are connected to the COPD methylation module are down-regulated, suggesting in most cases methylation represses transcription.

Pathophysiological mechanisms that may link fetal smoke exposure and adult COPD may be highlighted by the genes that connect the fetal lung methylation exposure module to the COPD modules. For example, *MAPK8* (a member of the fetal lung module which has connections to both COPD modules) which encodes the Mitogen-Activated protein kinase 8 (MAPK8) can be stimulated by environmental factors. Once MAPK8 is activated, it may target transcription factors that are involved in immediate early response [62–64]. *EGFR*, found in the COPD methylation module, encodes a transmembrane protein implicated in inflammation and airway remodeling [65, 66]. When activated, it mediates a signal transduction through the MAPK and JNK pathways. *BCL2*, a member of the COPD expression module, localizes to mitochondria [67] and regulates apoptosis through the release of cytochrome C and reactive oxygen species [68]. The BCL2 pathway can be regulated through the JNK pathway by phosphorylation and may impact immune responses [69–72]. BCL2 protein is increased in lung lymphocytes from smokers, which may influence chronic inflammation in COPD [73], and has been identified in COPD GWAS [74]. The gene *BCL2* has been identified as a key functional interactor with other COPD GWAS genes [37] through regulation of apoptosis and mitochondrial pathways [73, 75, 76]. While *MAPK8* and *EGFR* are located in the methylation modules, *BCL2* is located in the expression module but these genes are all connected to each other.

Interactor genes reveal the most robust enrichments and pathways between fetal IUS and COPD. Using the whole set of genes of a module (not only the interactors) the same or fewer pathways were enriched with limited statistical significance; thus, the results of the enrichment analysis did not improve. Also, no pathways were significantly enriched for the whole set of genes of the fetal lung methylation module, while three pathways were significantly enriched using only the interactors of this module. Seven pathways were significantly enriched using the whole set of genes of the COPD expression module, while using only the interactors gave rise to 13 significantly enriched pathways, including Focal Adhesion, AGE-RAGE, VEGF signaling pathway, and Pathways in cancer (Figs. 3, 4, Additional file 6: Table S5). Most of the genes in the pathways which were significantly enriched using the whole set of genes from the modules are interactors, further supporting the robust nature of the findings.

The identified pathways may link between perturbed lung development associated with exposure to cigarette smoke and COPD. The pathway which was significantly enriched for most gene sets (four out of seven gene sets) was the AGE-RAGE pathway, followed by the Focal-Adhesion pathway.

The AGE-RAGE pathway may be involved with COPD through inflammation [77, 78]. From a biomarker points of view, soluble receptor for advances glycosylation end products (RAGE) is the most compelling biomarker of adult COPD [79]. Given the role of the AGER-RAGE pathway in lung development and rodent models demonstrating links between maternal nicotine exposure and offspring perturbation of lung RAGE signaling [80, 81], we contend our method has identified biologically plausible pathways linking fetal lung perturbations and COPD. RAGE (encoded by *AGER*) has been implicated as a driver of cigarette smoke related emphysema [82], and circulating sRAGE has been implicated as a biomarker for emphysema [83]. *AGER* is not part of any of the three modules but is directly connected to the COPD expression disease module.

The Focal Adhesion pathway members facilitate physical links between the cytoskeleton of the cell to the extracellular matrix playing an important role in tissue organization and airway remodeling [84]. The AGE-RAGE and Focal Adhesion pathways are connected through *VEGFA*. The genes in the fetal lung methylation module are found up-stream in the AGE-RAGE pathway, whereas down-stream genes are from the COPD expression disease module. The up-stream part of Focal Adhesion pathway includes genes from the COPD methylation module and the COPD expression module genes are represented downstream. These pathways regulate closely related processes including airway inflammation and remodeling [77, 78, 84]. These findings require functional validation; however, we can speculate that this observation may represent a temporally directed relationship between the perturbed genes identified in the fetal lung and the genes related to COPD. Given the growing interest in targeting the AGE-RAGE pathway for lung disease our findings may suggest a future role for targeting the AGE-RAGE pathway for the primordial prevention of obstructive lung diseases.

Different approaches exist to identify network modules [85] and the focus in this current work is on PPI modules related to diseases. One main difference between the various approaches is that we are able to use published findings integrated in a network framework. Some approaches exploit only the topology of the PPI and employ knowledge from omic data sets afterwards to study the enrichment of the modules [17, 86–90]. Other methods use seed genes (5–30), genes that can be associated to a disease, and add new genes iteratively based on the topology of the network [34, 41, 91]. Another way to compute modules is to integrate omic data sets by using scores (e.g. p-values, fold change values, etc.) which are assigned to genes indicating their differential status in patients and control groups. Modules identified using omic data sets are called active modules [92] and there exist a variety of methods for computing these active disease modules, where most of them still rely on a set of seed genes as starting points [93]. Methods that are not using seed genes as a starting point are rare [94]; SigMod is most similar to our current method [95]. SigMod is based on optimization and computation of module scores, using p-values given by GWAS studies. The strategy favors high degree genes which are often genes which can be associated to diseases. However, even though some of the genes in our modules have a high degree in the underlying PPI, we do not explicitly favor these genes when using the ENCORe framework [42], since it computes modules which consist of genes which have small p-values and are highly connected to each other. Limitations of this approach include that the genes which are potentially crucial may be excluded from the module (like *AGER*) due to the p-value cutoff calculated by the method. However, we believe that using ENCORe provides us with a good balance between integrating scores on the genes based on disease affection status and the structure of the chosen PPI (Additional file 1 section “Disease modules integrating omic data sets”) (Additional file 7: Table S6, Additional file 8: Table S7, Additional file 9: Table S8).

Network-based approaches hold potential for studying fetal origins of complex lung diseases such as COPD [25–33]. Similar to the method we present, Halu et al. [25] computed a COPD disease module using a network-based approach and analyzed its vicinity to a pulmonary fibrosis disease module. Their modules for COPD and IPF are, like ours, significantly close to each other in the PPI and the biological pathways identified by Halu et al. give new potential insights into shared molecular interactions and shed light on biological processes lying at the intersection of these two incurable lung diseases. Maiorino et al. [27] introduce a method which calculates a ranking of genes linking two disease modules in a given PPI. They study genes linking a COPD disease module to an asthma disease module using the DIAMOnD approach [41]. They identified the asthma gene *GSDMB* and showed that by studying interconnecting genes it is possible to identify potential mediators of the interactions between different phenotypes. Both approaches [25, 27] use module detection methods based on seed genes and remaining module members are added solely based on the topology of the underlying PPI. Thus their methods differ profoundly from the method used in our work, and consequently the COPD modules have very different structures compared to the modules presented here.

## Conclusions

In utero exposures such as cigarette smoking and perturbed lung growth and development are associated with COPD, but there exists limited molecular links between early exposures, lung growth and adult disease. It is likely that in utero exposures do not impact single genes but networks of genes. Analyzing network connections between smoking-related perturbations during lung development and COPD is of clinical significance as identified genes and links may provide insights into biomarkers and targets for primary prevention of adult lung disease [58].

The modules identified in our analysis add new and potentially important insights and aspects to understanding the developmental pathogenesis of COPD. Strengths of our findings using ENCORe for the identification of biologically plausible pathways, including AGE-RAGE and focal adhesion, may reveal developmental contributions to COPD. Using ENCORe, we were not only able to identify meaningful modules but were also able to study possible relationships between early life exposure and adult lung phenotypes, thus augmenting our knowledge about the fetal origins of COPD.

## Supplementary Information

**Additional file 1: Figure S1.** The overlap of the significant genes from the different data sets. **Figure S2.** Schema for the approach. Based on a set of p-value cutoffs the method computes for each cutoff the largest connected component (LCC) given by all genes which have a p-value smaller than the cutoff. Next, for each LCC, its size (number of nodes) is compared against random expectation and a corresponding z-score is computed. The LCC with a z-score higher than 1.6 and containing genes with low p-values is considered to be the disease module. **Figure S3-S5.** The p-value cutoffs of the genes are given on the x-axis and the z-scores on the y-axis. For each p-value cutoff a LCC is computed using all genes of p-value lower than the cutoff. For this LCC a z-score is computed, using randomization. The z-scores are illustrated by the red dots. All details on the results can be found in the Table S8. **Figure S3.** Computation of the fetal lung methylation module. The module for the fetal lung methylation data set has a z-score of 2.86 at a p-value cutoff for the genes of 0.003. 265 genes in the data set have a p-value lower than this cut-off and they give a LCC of size 50, which is the exposure module for the fetal lung methylation data set. The size of the LCC given for all genes which have a p-value smaller than 0.01 is 289, therefore already too large for a reasonable disease module and therefore we did not consider higher p-value cutoffs. **Figure S4.** Computation of the COPD methylation module. The module for the COPD methylation data set has a z-score of 2.034 and the p-value cutoff for the genes is 0.037. 268 genes in the data set have a lower p-value than this cutoff and they give a LCC of size 37, which is the disease module for the COPD methylation data set. **Figure S5.** Computation of the COPD expression module. The module for the COPD expression data set has a z-score of 9.7 and is given by all genes which are significantly differentially expressed, thus which have a p-value lower than 0.05. They give a LCC of size 64, which is the disease module for the COPD expression data set. **Figure S6-S7.** Computation of the module using genes which are mapped to nominally differentially methylated CpG sites in both data sets: The p-value cutoffs of the genes are given on the x-axis and the z-scores on the y-axis. For each p-value cutoff a LCC is computed using all genes of p-value lower than the cut-off. For this LCC a z-score is computed, using randomization. The z-scores are illustrated by the red dots. All details on the results can be found in the Table S8. **Figure S6.** Using p-values from the fetal lung methylation data set: The module using p-values from the fetal lung methylation data set has a z-score of 3.2 at a p-value cutoff for the genes of 0.01. 202 genes in the data set have a p-value lower than this cut-off and they give a LCC of size 35. **Figure S7.** Using p-values from the COPD methylation data set: The module using p-values from the adult COPD patients methylation data set has a z-score of 2.2 at a p-value cutoff for the genes of 0.04. 248 genes in the data set have a p-value lower than this cut-off and they give a LCC of size 50. **Figure S8-S9.** Overlap modules: Using the 502 genes which are mapped to nominally differentially methylated CpG sites in the fetal lung methylation data set as well as in the COPD methylation data set we computed two modules using the p-values given by one of the data sets resp. The modules have 11 genes in common which are highlighted in red. **Figure S8.** Overlap module using fetal lung p-values: The module consists of 35 genes, where 11 of them can be found in the module constructed using the COPD p-values as well (highlighted in red). **Figure S9.** Overlap module using COPD p-values: The module consists of 50 genes, where 11 of them can be found in the module constructed using the fetal lung p-values as well (highlighted in red).

**Additional file 2: Table S1.** PropertiesDifferentPPIs: Properties of the different networks: We list here the properties of the networks we used for our analysis, where the HumanNet-FN was used for the main analysis. The networks are ordered by their size of the largest connected component. Network: Name of the network. Nodes: Number nodes in the network. Edges: Number of edges in the network. LCC Nodes: Number of nodes in the largest connected component of the network. LCC Edges: Number of edges in the largest connected component of the network. Website: website, where we downloaded the network (clickable). ConnectivityModulesInPPIs: Connectivity of modules in other PPIs: Using the genes of fetal lung methylation module and the two COPD modules we evaluatedconnectivity of the modules in the other PPIs. Network: The name ofnetwork. Fetal lung (50): The 50 genes of the fetal lung disease module were used for the analysis. COPD Meth (37): The 37 genes of the COPD methylation module were used for the analysis. COPD DE (64): The 64 genes of the COPD expression module were used for the analysis. LCC: The number of genes in the largest connected component (LCC) given by the genes ofdisease module. z-score: The z-score of the LCC in the network computed using the same number of nodes as in the disease modules randomly chosen from the network, where the degrees of the nodes were preserved. For example in the network BioGrid 32 genes of the fetal lung disease module (of sizeform a LCC. Thus 18 genes are not connected to this component. Note that HumanNet is the network where we computed the original modules.

**Additional file 3: Table S2.** The Table contains all the genes which are in the LCC of the HumanNet-FN.

**Additional file 4: Table S3.** Each list contains the genes within the corresponding module if they can be associated to respiratory diseases according to the database DisGeNet or GWAS study. Genes that can be associated to asthma and/or COPD according to DisGeNet are highlighted in green. Genes that can be associated to COPD according to GWAS are highlighted in yellow. Genes associated with asthma and COPD are highlighted in blue.

**Additional file 5: Table S4.** The table contains the genes of each module and their p-values as well as fold changes from the data sets when available.

**Additional file 6: Table S5.** The table ontains the results for the enrichment analyses using different sets of genes.

**Additional file 7: Table S6.** Results from enrichment analysis using g:profiler and the genes in the module compute using only genes which are mapped to nominally differentially methylated CpG sites in the fetal lung methylation data set as well as in the COPD methylation data set, using the p-values of the fetal lung methylation data set (sheet 1) and the p-values of the COPD methylation data set (sheet 2).

**Additional file 8: Table S7.** All genes and their degrees which are in one of the three modules. Their degrees in the subnetwork consisting of the three modules, the number of functional and physical edges connected to them and the corresponding p-values.

**Additional file 9: Table S8.** Details of the results using the method applied to the different data sets to compute the modules.

## Data Availability

All data analysed during this study are included in these published articles [and its Additional information files]: [36–38].

## Abbreviations

COPD: Chronic obstructive pulmonary disease
IUS: In utero smoke
PPI: Protein–protein interaction network
LCC: Largest connected component
GWAS: Genome-wide association studies
MAPK8: Mitogen-activated protein kinase 8
IPF: Idiopathic pulmonary fibrosis
