# Supplementary material for: Protein interaction networks provide insight into fetal origins of chronic obstructive pulmonary disease
Source: Respir Res. 2022 Mar 24;23:69. doi: 10.1186/s12931-022-01963-5 (PMC8944072; doi:10.1186/s12931-022-01963-5)
Supplement: Supplementary file 1 — Additional file 1: Figure S1. The overlap of the significant genes from the different data sets. Figure S2. Schema for the approach. Based on a set of p-value cutoffs the method computes for each cutoff the largest connected component (LCC) given by all genes which have a p-value smaller than the cutoff. Next, for each LCC, its size (number of nodes) is compared against random expectation and a corresponding z-score is computed. The LCC with a z-score higher than 1.6 and containing genes with low p-values is considered to be the disease module. Figure S3-S5. The p-value cutoffs of the genes are given on the x-axis and the z-scores on the y-axis. For each p-value cutoff a LCC is computed using all genes of p-value lower than the cutoff. For this LCC a z-score is computed, using randomization. The z-scores are illustrated by the red dots. All details on the results can be found in the Table S8. Figure S3. Computation of the fetal lung methylation module. The module for the fetal lung methylation data set has a z-score of 2.86 at a p-value cutoff for the genes of 0.003. 265 genes in the data set have a p-value lower than this cut-off and they give a LCC of size 50, which is the exposure module for the fetal lung methylation data set. The size of the LCC given for all genes which have a p-value smaller than 0.01 is 289, therefore already too large for a reasonable disease module and therefore we did not consider higher p-value cutoffs. Figure S4. Computation of the COPD methylation module. The module for the COPD methylation data set has a z-score of 2.034 and the p-value cutoff for the genes is 0.037. 268 genes in the data set have a lower p-value than this cutoff and they give a LCC of size 37, which is the disease module for the COPD methylation data set. Figure S5. Computation of the COPD expression module. The module for the COPD expression data set has a z-score of 9.7 and is given by all genes which are significantly differentially expressed, thus which have a p- [file 12931_2022_1963_MOESM1_ESM.doc]

# 1 Data

## 1.1 Fetal lung

The fetal lung DNA samples included 78 fetal lung samples that passed the quality control measures [36]. Methylation in smoke-exposed was compared to unexposed fetal lung samples and were considered nominally significant at a p-value cut off of 0.05. The fetal lung DNA samples were isolated from discarded tissue from 8-18 weeks of gestation. The samples were anonymized at study entry at the Laboratory of Developmental Biology, University of Washington, Seattle, WA, USA.

Genome-wide methylation assay was performed using 750 ng of bisulfite-treated DNA per sample using the Infinium HumanMethylation450 BeadChip array (Illumina, San Diego, CA, USA), according to manufacturer’s recommended protocol. Data were available for gestational age, fetal sex, and cotinine levels. Sex was verified using X chromosome methylation. IUS exposure was inferred by measuring placental cotinine concentrations. Exposure was treated as a continuous and dichotomous variable, with levels of cotinine ≤ 7.5 ng/g considered as unexposed (control group) and levels of cotinine > 7.5 ng/g as exposed. Site based differential methylation analysis was performed using linear regression models as a part of Bioconductor package limma (version 3.37.7) [39] and minfi adjusting for age, sex, sample plate, and sentrix position. DM sites were categorized as relatively hyper- or hypo-methylated if they showed higher or lower percent methylation in smoke-exposed compared to unexposed fetal lung samples respectively and were considered nominally significant at a p-value cut off of 0.05. CpG sites were mapped to genes using Human Genome build: GRCh37/hg19 annotation.

## 1.2 COPD

Genome-wide methylation assay was performed using 750 ng of bisulfite-treated DNA per sample using the Infinium HumanMethylation450 BeadChip array (Illumina, San Diego, CA, USA), according to manufacturer’s recommended protocol [37, 38]. The study included lung tissue samples from 114 COPD cases and 46 control smokers with normal lung function. Site based differential methylation and expression analysis was performed using linear regression models as a part of Bioconductor package limma (version 3.37.7) [40] and minfi adjusting for race (C, AA, Others), sex, and sample plate. DM sites were categorized as relatively hyper- or hypo-methylated if they showed higher or lower percent methylation

in smoke-exposed compared to unexposed fetal lung samples respectively and were considered nominally significant at a p-value cut off of 0.05. CpG sites were mapped to genes using Human Genome build: GRCh37/hg19 annotation.

## 1.3 Significant genes

In total 5,175 genes are mapped to nominally diﬀerentially methylated CpG sites in the fetal lung data set [36], 1,217 in the COPD data set, and 204 genes were differentially expressed in the COPD data set [37, 38] (p-value < 0.05). More details can be found in Table 1 and Figure S1.

# 2 Computation of the modules

Typically, disease genes (DGs) are no hubs, resp. essential genes. However, their average connectivity is higher than for an ordinary gene although they avoid dense clustering [41, 95]. Therefore, we used the network-based method of Wang et. al [42] which computes modules based on the connectivity of the genes contained within the modules while integrating scores based on disease affection status (e.g. p-values or fold change values). The framework identifies disease modules by agglomerating genes based on their significance within their respective study.

The original approach by Wang et. al ranks in a first step all significantly differentially expressed genes according to their fold change value. For our purposes we rank them by their p-values. All remaining steps of the method are the same as in [42] and are explained in detail in the following:

As mentioned above, we first rank genes according to their p-value from the most significant to the least significant (low p-value to high p-value, up to a p-value of 0.05). Next, we define different p-value thresholds. For each p-value threshold the LCC given by all genes which have a lower p-value than the current threshold is computed. The size of each LCC is compared against random expectation, resulting in a corresponding z-score. This procedure results in a p-value threshold vs. z-score plot which is used to determine the lowest p-value cutoff that has a high z-score (above the minimum requirement of 1.6). The resulting module consists of the LCC which is given by all genes with p-values below this threshold. Hence, the method ensures that the most strongly implicated genes are preferentially added to the module while significant module connectivity is maintained. An illustration of the approach is shown in Figure S2.

The method can be divided into five steps:

**Step 1** Order genes according to their p-values: Each gene has a given p-value and the genes are ranked accordingly: The highest rank gene has the smallest p-value and the lowest ranked gene has the highest p-value.

**Step 2** Compute LCCs for given p-value cutoffs: For a given number of p-value cutoffs, between 30 and 50, we perform for each cutoff the following procedure, starting from the lowest p-value cutoff: use the set of genes which have a p-value lower than the current cutoff and compute the corresponding LCC in the PPI. Next, increase the cutoff. Again, compute the corresponding LCC for the new given cutoff. Repeat this step, until the highest cutoff is reached (typically a p-value of 0.05, since we only

want to consider significant genes).

**Step 3** Compute z-score for the LCC: For each LCC computed in Step 2 we calculate a z-score by comparing the size of the LCC to what is expected by chance. We perform the procedure of the second step 1,000 times, each time using a set of genes of the same size as given by the p-value cutoff but randomly chosen from the set of all genes in the PPI. Doing so, we can compute the average size of the LCCs given by a set of genes of this size and the standard deviation. The z-score of the LCC is then given as:

z−score = (|LCC| − rand(|LCC |)) /std(|LCC |)

where |LCC| denotes the size of the LCC computed in the second step, rand(|LCC|) denotes the average size of an LCC induced by a set of genes of same size as given by the cutoff from Step 2, and std(LCC|) is the standard deviation of the size of the LCCs. (Note: To save running time, computing the average size and standard deviation of the size of a LCC for a given set of genes was done before the actual computing and stored in a file.)

**Step 4** Disease module: The LCC with a z-score higher than 1.6 and containing genes with low p-values is considered to be the disease module.

## 2.1 Computation of the fetal lung methylation module

We applied the method described above to the fetal lung methylation data set. The p-value cutoff for the genes in the exposure module was 0.003. In total 265 genes have a p-value lower than this cutoff in the whole data set and the given LCC is of size 50 with a z-score of 2.86 (Figures 1b and S2).

## 2.2 Computation of the COPD methylation module

We applied the method to the COPD methylation data set. The p-value cutoff for the genes in the computed module was 0.037. In total 268 genes have a p-value lower than this cutoff in the whole data set and the given LCC is of size 37 with a z-score of 2.034 (Figures 1c and S3).

## 2.3 Computation of the COPD expression module

We applied the method to the COPD expression data set. The p-value cutoff for the genes in the computed disease module was 0.05, thus all significantly differentially expressed genes are used to identify the LCC, and therefore the disease module, which is a connected component of of size 64 with a z-score of 9.7 (Figures 1d and S4).

## 2.4 Computation of the modules using genes which are significantly enriched in both methylation data sets

We applied the method onto the 502 genes which are mapped to nominally diﬀerentially methylated CpG sites in the fetal lung data set as well as in the adult COPD patient data set. Since the method uses as input a set of p-values we computed two modules, one using the p-values given by the fetal lung methylation data set (Figures S5), the other module given by the p-values by the adult COPD patient methylation data set (Figures S6). The p-value cutoff for the genes in the computed module using p-values given by the fetal lung methylation data set is 0.01 and consists of 35 genes, whereas the cutoff for the genes in the module computed using the p-values given by the COPD methylation data set is 0.04 and the module consists of 50 genes (Figure S7). The modules have 11 genes in common, namely: PRKAR1B, LAMA4, NCOR2, KNDC1, HDAC4, SKI, NRXN3, RUNX3, CHRM1, RUNX1, and SHANK2.

Next, we performed an enrichment analysis on each of the two sets of genes from the two modules using the LCC given by the HUmanNet-FN as a background. The results for performing an enrichment analysis on these modules were very sparse. Using the genes of the module constructed using the p-

values from the COPD methylation data set, biological processes related to the development of cells and organs were enriched, pointing to the potential effect of smoking on lung development and the potential risk of the manifestation of COPD (Table S6).

# 3 Details on the modules

Details on the genes and their associations to diseases can be found in tables 3 and S2. The degrees of the genes in the HumanNet-FN as well as in the modules can be found in the Table S7.

## 3.1 Fetal lung methylation module

The set of 5,175 genes are mapped to nominally diﬀerentially methylated CpG sites in the fetal lung methylation data set produces an exposure module which consists of 50 genes, where seven of the genes are also are mapped to nominally diﬀerentially methylated CpG sites in the COPD data set and one is differentially expressed (Figure 1b). We found that 7 of the 50 genes can be associated to COPD, including 7 out ofthe 14 interacting genes (highlighted in bold): AGTR1, **AKAP5**, AP2A2, **CCR5**, DNMT3B, **EEF1A2**, **HDAC3**, **IL1RN**, **MAPK8**, **MDK**, OBSCN, and ZFPM1.

There are several interesting properties which can be observed for these genes.

For example MAPK8 is a connecting gene to the COPD methylation disease module, cannot be associated to COPD but to asthma according to DisGeNet.

However, MAPK8 is connected to IL1RN and CCR5, which can be both related to COPD and asthma, and CCR5 again is connected to MDK which is also related to COPD. Therefore, we have a small connected component, consisting of four genes which are all related to COPD in different ways. One gene, or step, away from this connected component are EEF1A2 and HDAC3, where EEF1A2 is are mapped to nominally diﬀerentially methylated CpG sites for COPD (although it is not part of the COPD methylation disease module) and can be related to asthma, while HDAC3 can be associated to COPD too and is connected to BCL11B, a gene which can be mapped to nominally diﬀerentially methylated CpG site(s) from the COPD data set.

## 3.2 COPD methylation disease module

The COPD methylation disease module given by the 1,217 genes which are mapped to nominally diﬀerentially methylated CpG sites in the adult COPD data set (adj. P-value < 0.05) [37] consists of 37 genes, where no gene is significantly differentially expressed in the COPD data set (Figure 1c). The following three genes can be associated to COPD: EGFR, EPHB2, and PARVA (Figure 1c and Table 3).

Especially EGFR and ERBB2 seem to play an important role. Both connect the COPD methylation disease module to the fetal lung methylation module and EGFR is related to 36 respiratory diseases, including COPD, asthma, and different lung diseases, while ERBB2 is related to 14 respiratory diseases, including asthma and different lung diseases (but not COPD). Other interactors which are related to asthma are ETS1, CHRM1, and CEBPA. Interactors, which are related to lung diseases are EGFR, ERBB2, ETS1, CEBPA, PXN, DAPK1, DAPK2, and RUNX1T1.

## 3.3 COPD expression module

There are 204 genes significantly differentially expressed in the adult COPD data set (adj. p-value < 0.05) [37] and the given disease module consists of 64 genes (Figure 1d). 11 genes of the module can be associated to COPD.

## 3.4 Modules computed using genes which are significantly enriched in both methylation data sets

We applied the method onto the 502 genes which are significantly differentially methylated in the fetal lung data set as well as in the adult COPD patient data set. Since the method uses as input a set of p-values we computed two modules, one using the p-values given by the fetal lung methylation data set,

the other module given by the p-values by the adult COPD patient methylation data set (Figure S9). The computed module using p-values given by the fetal lung methylation data set consists of 35 genes, whereas the module computed using the p-values given by the COPD methylation data set consists of 50 genes.

The modules have 11 genes in common, namely: PRKAR1B, LAMA4, NCOR2, KNDC1, HDAC4, SKI, NRXN3, RUNX3, CHRM1, RUNX1, and SHANK2 (Figure S9).

Next, we performed an enrichment analysis on each of the two sets of genes from the two modules using the LCC given by the HUmanNet-FN as a background. The results for performing an enrichment analysis on these modules were very sparse. Using the genes of the module constructed using the p-values from the COPD methylation data set biological processes related to the development of cells and organs were enriched, pointing to the potential effect of smoking on lung development and the potential risk of the manifestation of COPD (Table S6).

# 4 Connectivity between the modules

In order to analyze if the connectivity between the modules is topologically significant we compared the number of connections between the modules to what is expected by chance.

In a first step we compute modules which are of the same size as the three modules in our study: We identify the *critical* genes within these modules w.r.t. their connectivity: We compute for each gene a z-score comparing the degree of the gene to the other genes within these modules:

z − score = (d(g) − d_mean_) / d_std_

where d(g) is the degree of gene g within the module, d_mean_ is the average degree of the genes within the module, and d_std_ is the standard deviation of the degree of the genes within the module.

All genes which have a z-score larger than 1.6 have a significantly high degree within this module. Therefore, we keep these genes and construct around them a new module by choosing genes randomly from the PPI until we have a new module of the same size as the original one. We compute for each of the three modules (fetal lung methylation, adult COPD patients methylation, and adult COPD patients expression modules) 1,000 of these randomized modules.

Next, we calculate the number of connections between these randomized modules and the average number and standard deviation of the number of connections between these modules. Using these numbers we can again compute z-scores regarding the significance of the connectivity between the three original modules.

The z-score regarding the connectivity between the fetal lung methylation module and the adult COPD patient methylation module is 23.8 and between the fetal lung methylation module and the adult COPD patient expression mod ule is 17.46. Therefore, the vicinity between the modules is significantly higher

than by chance, even if we build the randomized modules around the hubs of the original modules.

## 4.1 Connectivity of the genes which can be associated to COPD

In order to compute the significance of the size of the LCC given by the genes which can be associated to COPD in the subnetwork given by all three modules, we compare its size to what is expected by chance. We use a set of genes of the same size as the set of genes which can be associated to COPD in the subnetwork but randomly chosen from the connected component given by the three modules and repeat this 1,000 times. Doing so, we can compute the average size of the LCCs given by a set of genes of this size and the standard deviation. The z-score of the LCC is then given as:

z−score = (|LCC| − rand(|LCC |)) /std(|LCC |)

where |LCC| denotes the size of the LCC computed in the second step, rand(|LCC|) denotes the average size of an LCC induced by a set of genes of same size as the set of genes which can be associated to COPD in the subnetwork, and std(LCC|) is the standard deviation of the size of the LCCs.

# 5 Robustness

In the following we show the robustness of the computed modules. We analyze the connectivity of the modules in different PPIs to evaluate their robustness within other PPIs. The details of the networks can be found in the Table S1.

To analyze the robustness of the three modules computed using the approach of Wang et. al [42] and the HumanNet-FN, we studied the size of the three modules in five other PPIs (BioGRID [43], STRING [44], Hint [45], PPI2016 [46], and BioPlex [47]). We computed the size of the LCC given by the genes of the modules and the average distance of the genes which are not contained in the LCC.

Additionally, we compute a z-score regarding which size of LCC is expected when using the same number of genes as in the module. We compute 1,000 LCCs given by the same number of genes which are chosen randomly, but degree persevered, from the PPI to compute a z-score (Step 3 in Supplementary Section 2).

The results (Table S1) show that the sparser the network is the smaller are the LCCs given by the genes in the disease module, which supports the hypothesis that modern PPIs are still missing edges, i. e. there exist unknown interactions between genes. We also compute the average and shortest distance of genes which were not in the LCCs to the modules within the corresponding networks (Table S1)

# 6 Related work

There exist different approaches to identify communities or modules in networks [86] and the focus in this work lies on modules in PPIs related to diseases. One main difference between the various approaches is which data is used and how this data is integrated. We consider here three different categories. The first type of approaches exploit only the topology of the network to compute modules

and apply omic data sets later to study the enrichment of the modules. The methods in the second category use seed genes (disease genes), which are usually only 30 to 100 genes, not taken into account their p-values or any other scores. The last category uses omic data sets where scores (e.g. p-values, fold change values, etc.) are assigned to genes indicating their different status in patients and control groups.

## 6.1 Disease module computation using topology only

Different approaches exist which rely on the topology of a given interactome only. Several methods are based on maximizing the modularity [87, 88, 89], where genes are assigned to the community which gains most value by obtaining this gene as a member. Other methods are based on network diffusion [97], resp. label propagation where each gene is initiated with a unique label (e.g. its degree) and at every step each gene is assigned the label most of its neighbors have. The approaches mentioned above all compute non-overlapping modules.

The method of [90] computes overlapping modules based on edge-similarity and [91] based on cohesiveness of the genes in the network w.r.t. to a seed gene which is the gene with the highest degree.

## 6.2 Computing disease modules using seed genes

In 2015, Ghassian et. al introduced a Disease Module Detection (DIAMOnD) algorithm [41], which identifies the disease neighborhood around a set of known disease genes based on connectivity significance and which was applied successfully to identify disease modules [17].

In 2018 Vlaic et. al presented ModuleDiscoverer [92], a method which uses a randomization heuristic-based approximation of the community structure and, like DIAMOnD, the method uses seed genes. Their approach can be divided into three steps: 1) Compute minimal cliques of size three which include the seed genes. Extend these cliques until not further possible. (The fewer seed genes are used the larger the cliques, thus small cliques and their genes can be missed.

More seed genes result in smaller cliques, thus more genes are involved, which may include non DEGs too and therefore add noise to the result), 2) identify highly enriched cliques and compute their p-value, 3) identify significant cliques.

In 2011, Erten et. al introduced in [34] DADA, a suite which includes different prioritization methods, where several statistical adjustments strategies are added since gene prioritization is highly depended on the degree distribution of the PPI. The prioritization methods they consider are all so-called global methods (i.e. they take the whole network into account and not only the neighborhood of the seed genes) and are based on random walk with restarts and network propagation. All the methods they improved by their statistical adjustments are based on seed genes, i.e. disease genes.

The main difference between the method used in this work and the three methods above (DIAMOnD, ModuleDiscoverer, and DADA) is that the other approaches start with a set of disease genes (seed nodes) and add genes accordingly. Therefore, no scores for genes (e.g. p-values) are included and the result depends highly on the set of disease gene which are chosen in the beginning.

Modules computed exploiting the topology of the network only do not integrate omic data, e.g. gained from experiments as it is done by the method used in this work. Modules identified using omic data sets are called active modules and we will consider methods to compute them in the following.

## 6.3 Disease modules integrating omic data sets

There exist a huge variety for computing active disease modules (disease modules identified integrating omic data sets) [34], where most of them still rely on a set of genes as starting points, thus seed genes [94, 63, 64, 66].

The method of Guo et. al [67] uses transcriptomic data to weight the edges of the underlying PPI: the weight of an edge is based on the Pearson correlation coefficient of the expressions of the genes which the edge is connecting. Their algorithm starts by choosing a set of edges randomly. Next, they compute all given LCCs by the edges and the scores of the LCCs given by the weights of the edges. Given a pre-defined number of iterations they add in each step a randomly chosen edge and calculate the score the new LCC. Finally, the LCC of highest score is chosen. Compared to our method a random set of interactions, and therefore genes, is chosen in the beginning. The method we use chooses genes, and subnetworks, given by the genes’ p-values and the z-score of the LCCs given by the genes. Therefore, the subnetworks we computed are based solely on the genes’ p-values and the topology of the PPI and not on chance, which we believe is an advantage.

Another method which computes active modules without making use of seed genes is *SigMod*, the method introduced in [69]. This is probably the method most similar to ours. Their method is based on optimization and they compute a score for their modules as well. They start with assigning weights to genes based on their p-values given by GWAS studies. Next, they solve an optimization problem where the sub-network with highest connectivity and highest scored genes is identified. Their strategy however favors high degree genes which they state are often genes which can be associated to diseases. However, even though some of the genes in our modules have a high degree in the underlying PPI we do not explicitly favor these genes when using the method of Wang et. al [42]. We believe that using the method of [42] provides us with a good balance between integrating scores on the genes based on disease affection status and the structure of the chosen PPI.

# 7 Hypergeometric p-value

In order to compute the chances to find genes in the modules which can be associated to COPD we compute the hypergeometric p-value:

The hypergeometric distribution gives the probability of *k* successes in n draws (without replacement) from a total population of size *N*, where *K* objects are considered as being a success. The probability that a drawn sample consists of *k* successes can be computed using:


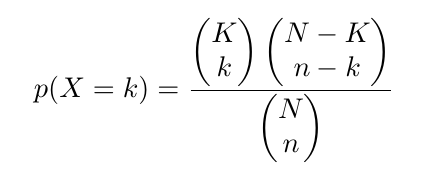


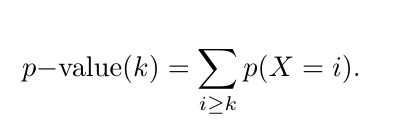
*where X* is a random variable. The corresponding p-value can then be computed using the following formula:

To compute the hypergeometric p-value of having *k* genes which can be associated to COPD in a module of size *n,* we set *K* = 1,245, which is the number of all genes in the HumanNet-FN which can be associated to COPD, and *N* = 17,247, the total number of genes in the HumanNet-FN.

# 8 Captions Figures

**Figure S1:** The overlap of the significant genes from the different data sets.

**Figure S2: Schema for the approach.** Based on a set of p-value cutoffs the method computes for each cutoff the largest connected component (LCC) given by all genes which have a p-value smaller than the cutoff. Next, for each LCC, its size (number of nodes) is compared against random expectation and a corresponding z-score is computed. The LCC with a z-score higher than 1.6 and containing genes with low p-values is considered to be the disease module.

**Figure S3-S5:** The p-value cutoffs of the genes are given on the x-axis and the z-scores on the y-axis. For each p-value cutoff a LCC is computed using all genes of p-value lower than the cutoff. For this LCC a z-score is computed, using randomization. The z-scores are illustrated by the red dots. All details on the results can be found in the Table S8.

**Figure S3: Computation of the fetal lung methylation module.** The module for the fetal lung methylation data

set has a z-score of 2.86 at a p-value cutoff for the genes of 0.003. 265 genes in the data set have a p-value lower than this cut-off and they give a LCC of size 50, which is the exposure module for the fetal lung methylation data set. The size of the LCC given for all genes which have a p-value smaller than 0.01 is 289, therefore already

too large for a reasonable disease module and therefore we did not consider higher p-value cutoffs.

**Figure S4: Computation of the COPD methylation module.** The module for the COPD methylation data set has a z-score of 2.034 and the p-value cutoff for the genes is 0.037. 268 genes in the data set have a lower p-value than this cutoff and they give a LCC of size 37, which is the disease module for the COPD methylation data set.

**Figure S5: Computation of the COPD expression module.** The module for the COPD expression data set has a z-score of 9.7 and is given by all genes which are significantly differentially expressed, thus which have a p-value lower than 0.05. They give a LCC of size 64, which is the disease module for the COPD expression data set.

**Figure S6-S7: Computation of the module using genes which are mapped to nominally diﬀerentially methylated CpG sites in both data sets:** The p-value cutoffs of the genes are given on the x-axis and the z-scores on the y-axis. For each

p-value cutoff a LCC is computed using all genes of p-value lower than the cut-off. For this LCC a z-score is computed, using randomization. The z-scores are illustrated by the red dots. All details on the results can be found in the Table S8.

**Figure S6: Using p-values from the fetal lung methylation data set:** The module using p-values from the fetal lung methylation data set has a z-score of 3.2 at a p-value cutoff for the genes of 0.01. 202 genes in the data set have a p-value lower than this cut-off and they give a LCC of size 35.

**Figure S7: Using p-values from the COPD methylation data set:** The module using p-values from the adult COPD patients methylation data set has a z-score of 2.2 at a p-value cutoff for the genes of 0.04. 248 genes in the data set have a p-value lower than this cut-off and they give a LCC of size 50.

**Figure S8-S9:Overlap modules:** Using the 502 genes which are mapped to nominally diﬀerentially methylated CpG sites in the fetal lung methylation data set as well as in the COPD methylation data set we computed two modules using the p-values given by one of the data sets resp. The modules have 11 genes in common which are highlighted in red.

**Figure S8: Overlap module using fetal lung p-values:** The module consists of 35 genes, where 11 of them can be found in the module constructed using the COPD p-values as well (highlighted in red).

**Figure S9: Overlap module using COPD p-values:** The module consists of 50 genes, where 11 of them can be found in the module constructed using the fetal lung p-values as well (highlighted in red).

# 9 Captions Tables

**Table S1:**

**PropertiesDifferentPPIs:** Properties of the different networks: We list here the properties of the networks we used for our analysis, where the HumanNet-FN was used for the main analysis. The networks are ordered by their size of the largest connected component. Network: Name of the network. Nodes: Number nodes in the network. Edges: Number of edges in the network. LCC Nodes: Number of nodes in the largest connected component of the network. LCC Edges: Number of edges in the largest connected component of the network. Website: website, where we downloaded the network (clickable).

**ConnectivityModulesInPPIs:** Connectivity of modules in other PPIs: Using the genes of fetal lung methylation module and the two COPD modules we evaluatedconnectivity of the modules in the other PPIs. Network: The name ofnetwork. Fetal lung (50): The 50 genes of the fetal lung disease module were used for the analysis. COPD Meth (37): The 37 genes of the COPD methylation module were used for the analysis. COPD DE (64): The 64 genes of the COPD expression module were used for the analysis. LCC: The number of genes in the largest connected component (LCC) given by the genes ofdisease module. z-score: The z-score of the LCC in the network computed using the same number of nodes as in the disease modules randomly chosen from the network, where the degrees of the nodes were preserved. For example in the network BioGrid 32 genes of the fetal lung disease module (of sizeform a LCC. Thus 18 genes are not connected to this component. Note that HumanNet is the network where we computed the original modules.

**Table S2:**

The Table contains all the genes which are in the LCC of the HumanNet-FN.

**Table S3:**Each list contains the genes within the corresponding module if they can be associated to respiratory diseases according to the database DisGeNet or GWAS study. Genes that can be associated to asthma and/or COPD according to DisGeNet are highlighted in green. Genes that can be associated to COPD according to GWAS are highlighted in yellow. Genes associated with asthma and COPD are highlighted in blue.

**Table S4:**

The table contains the genes of each module and their p-values as well as fold changes from the data sets when available.

**Table S5:**

The table ontains the results for the enrichment analyses using different sets of genes.

**Table S6:** Results from enrichment analysis using g:profiler and the genes in the module compute using only genes which are mapped to nominally diﬀerentially methylated CpG sites in the fetal lung methylation data set as well as in the COPD methylation data set, using the p-values of the fetal lung methylation data set (sheet 1) and the p-values of the COPD methylation data set (sheet 2).

**Table S7:** All genes and their degrees which are in one of the three modules. Their degrees in the subnetwork consisting of the three modules, the number of functional and physical edges connected to them and the corresponding p-values.

**Table S8:** Details of the results using the method applied to the different data sets to compute the modules.
